# Age and sex differences in comorbidities in adult temporomandibular disorders: A cross-sectional study using Korea National Health and Nutrition Examination Survey (KNHANES)

**DOI:** 10.1371/journal.pone.0296378

**Published:** 2024-01-02

**Authors:** Hye-Ji Park, Q-Schick Auh

**Affiliations:** 1 Department of Oral Medicine, Kyung Hee University Hospital at Gangdong, Seoul, Korea; 2 Department of Oral Medicine, Kyung Hee University College of Dentistry, Kyung Hee University Medical Center, Seoul, Korea; University of Catanzaro, ITALY

## Abstract

**Objectives:**

To investigate the relationship between Temporomandibular disorder (TMD) and associated comorbidities in groups matched according to age and sex.

**Methods:**

Using data from the cross-sectional fifth Korea National Health and Nutrition Examination Survey (KNHANES). Of the 25,534 eligible KNHANES, 17,762 adults aged ≥19 years who responded to survey questionnaire on TMD and comorbidities. Subjects were classified into eight groups according to age and sex. Logistic regression analyses were performed to evaluate the association between TMD and comorbidities according to age and sex.

**Results:**

Of the enrolled participants, 2,107 (11.86%) complained of ≥1 TMD symptoms. In all groups, odds ratios (ORs) for prevalence of TMD were >1 in those with tinnitus. Rhinitis was closely associated with TMD in 6 groups. ORs for TMD with comorbidities according to age and sex were as follows: hypertension, men aged 50–64 years (OR 0.62; CI 0.41–0.94); ischemic heart disease, men aged 35–49 years (4.38; 1.54–12.47); osteoarthritis, women aged 50–64 years (1.38; 1.03–1.86); diabetes mellitus, men aged 35–49 years (0.21; 0.05–0.88); depression, men aged 50–64 years (1.68; 1.00–2.83), women aged 35–49 years (1.39; 1.05–1.85) and women aged 65–80 years (2.01; 1.46–2.77); migraine, men aged 50–64 years (1.60; 1.14–2.25), women aged d35-49 years (1.44; 1.14–1.81) and women aged 35–49 years (1.43; 1.07–1.90); cold hypersensitivity in the hands and feet, men aged 19–34 years (1.64; 1.05–2.58), men aged 35–49 years (1.68; 1.04–2.70), men aged 65–80 years (1.74; 1.09–2.75) and women aged 35–49 years (1.45; 1.15–1.84); olfaction disorder, men aged 50–64 years (2.49; 1.39–4.43); voice disorder, men aged 50–64 years (2.25; 1.28–3.96) and women aged 65–80 years (1.69; 1.09–2.63)

**Conclusions:**

This study confirmed that the types and effects of comorbidities related to prevalence of TMD may differ according to the patient’s age and sex and this result will increase the predictability of the onset of TMD.

## Introduction

Temporomandibular disorders (TMD) encompass a range of musculoskeletal diseases of orofacial region that impact the masticatory muscles and temporomandibular joint (TMJ) [1]. The main clinical features of TMD is persistent or repetitive pain in the TMJ, masticatory muscles and surrounding tissues, decreased range of motion of the mandible and joint noise [2]. The prevalence of TMD is estimated to range from 21.1% to 73.3% in the general population, and these conditions are most common in women of childbearing age [3]. The prevalence of TMD peaks between the ages of 20 and 40 years, and can occurs in about 20–60% of children and adolescents [4]. In particular, assuming that female hormones affect a high prevalence of TMD in women group regardless of ages, the prevalence comparison between pregnant and non-pregnant women was indirectly confirmed, however there was no significant difference between those groups [5]. Recently, the Korea National Health Insurance Service announced that approximately 410,000 South Koreans visited a hospital due to TMD in 2019, an increase of approximately 17% as compared to 2015. In addition, it has been reported that approximately 3.1% of patients with TMD have chronic symptoms that persist for ≥3 months [6].

Among the symptoms of TMD, pain is associated with other chronic pain disorders and physical symptoms present within comorbidities, including joint and neck muscle pain [7]. In the past, there was a tendency to view the symptoms of individuals complaining of pain in various areas as merely psychosocial (i.e., psychosomatic) problems [8]. Currently, researchers and physicians interpret findings of persistent pain in various regions as potentially due to dysfunction of the nociceptive nervous system.

Patients complaining of headache, low back pain, and neck pain tend to present with signs and symptoms relevant to TMD, which are also associated with sleep disturbance, forgetfulness, and irritable bowel syndrome [3]. The origin of this phenomenon is not yet clear. However, this phenomenon is frequently reported in combination with neurobiological sensitization processes, genetic vulnerabilities, and psychosocial factors [9]. To elucidate the mechanisms underlying TMD, it is important to study associated comorbidities in combination such that shared influencing factors for TMD and comorbidities can be determined.

Although there have been many previous studies on comorbidities within TMD, one question that has not been addressed within prior research is whether there are age and sex differences in TMD-associated comorbidities. TMD does not occur uniformly in all ages and sex and there is a clear difference between the age and sex of the predisposition. In general, women with TMD are more common than men and the prevalence of TMD symptoms has traditionally been thought to peak between the ages of 20 and 40, with younger and older age groups having a lower prevalence [10]. Recent research has revealed two unique maxima in patient populations for particular TMD conditions: one over 50 years old for inflammatory-degenerative joint disorders and the other around 30 years old for subjects with disc displacements [11]. This means that major factors influencing the onset of TMD may be different when age and sex are adjusted. Predicting the onset of TMD would be much easier if the different major factors and their influence could be identified for a given age and sex.

When comparing the two groups, controlling for the effects of basic characteristic variables such as age and sex and examining differences in clinical variables between groups with secured equivalence can enhance clinical explanatory power and facilitate clinical interpretation. In other words, if the test and control groups are not homogeneous in terms of basic characteristic variables, the results may be underestimated or overestimated and selection bias may occur [12]. Of course, there are statistical corrections to solve this problem, but it is important to secure homogeneity between the two groups, except for the variables to be compared in the first place.

This study aimed to investigate the relationship between TMD and comorbidities within groups matched by age and sex using large-scale data from the 5^th^ Korea National Health and Nutrition Examination Survey (KNHANES V). In addition, we can expect which comorbidities may be a substantial contributor for the onset of TMD according to age and sex.

## Methods

### Study population and group

This study was conducted within KNHANES V (2010–2012). The KNHANES is a nationwide review that evaluates the general health and nutrition status of South Koreans and offers policy references informing public health programming. Three years of survey information for 11,520 households in 576 sampling districts was collected using a complex, stratified, multistage, probability-cluster design. Approximately 26,000 individuals participated in this survey. Analyses were conducted in accordance with policies on the approved use of raw data originating from the KNHANES V delineated by the Korea Centers for Disease Control and Prevention (KCDC). Additional information on KNHANES V methodology is available at http://knhanes.cdc.go.kr.

**Fig 1:**
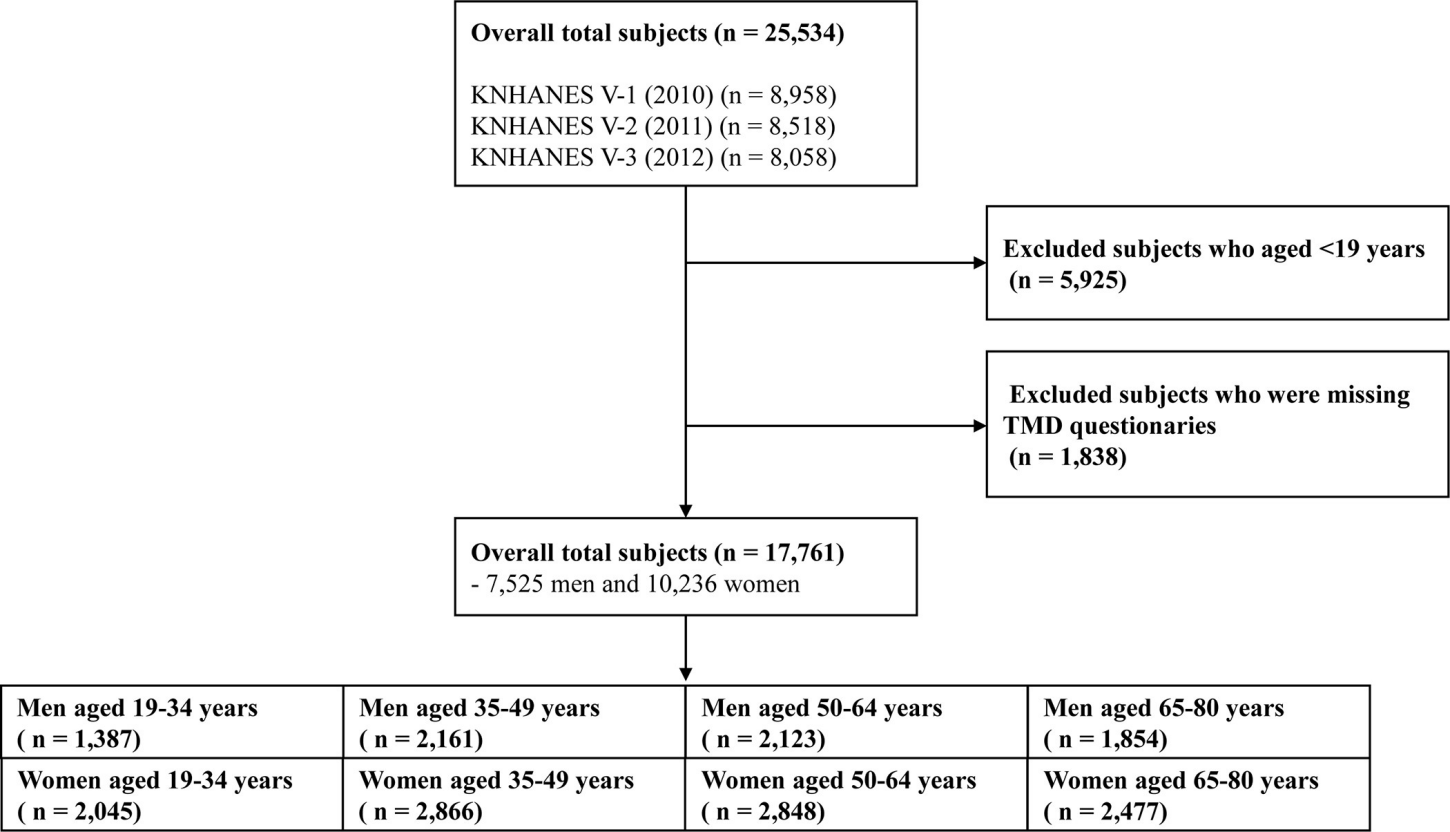
Participant’s flowchart.

This was a retrospective, cross-sectional study. Among the 25,534 eligible participants in KNHANES V, we excluded 5,935 participants aged <19 years. In addition, we excluded 1,838 participants who were missing TMD symptom questionnaires. A total of 17,761 participants (7,525 men and 10,236 women) were included in the present study (Fig 1). In this survey, subjects were divided into eight groups with matching according to sex (men and women) and age (19–34, 35–49, 50–64, and 65–80 years). Referring to the age classification criteria for each life cycle of the Korea Institution for Health and Social Affairs (early adulthood 19–34 years, early middle age 35–49 years, late middle age 50–64 years, late adulthood 65 years or older) [13], the age of our study population was divided into the above four groups. The criteria for dividing the age into 5 and 10 units also exist, but the fact that statistical power may be lost due to over segmentation of groups.

### Outcome and other variables

#### Assessment of TMD symptoms

For the TMD evaluation, subjects marked “yes” (indicative of experience of the referenced symptoms at least once a week) within the following three questions suggested by the World Health Organization [14]:

1. Clicking sounds in one or both auricular areas.
2. Symptoms of tightness or pain in one or both ear regions, temples, or cheek areas.
3. Pain or discomfort when opening the mouth, reduced jaw mobility, or a dislocated jaw.

A trained dentist performed clinical and TMD examinations to confirm the questionnaire items. Patients with TMD were included in the study sample if one or more of the above three items were present.

#### Assessment of comorbidities

The prevalence of the following comorbidities was evaluated according to individuals’ histories of the following conditions as indicated within the survey: hypertension, ischemic heart disease, stroke, dyslipidemia, liver cirrhosis, hepatitis C, hepatitis B, renal insufficiency, tuberculosis, diabetes mellitus, thyroid diseases, asthma, atopic dermatitis, rheumatoid arthritis, osteoarthritis, migraine, cold hypersensitivity in the hands and feet(CHHF), depression, tinnitus, otitis media, rhinitis, allergic rhinitis, sinusitis, olfaction disorder and voice disorder.

#### Assessment of correlation

Of the many survey items available within KNHANES V, our study included sex, age, town, household income, education, occupation, and marital status for social-demographic characteristics. The town variable was categorized according to administrative district. Dong is typically the administrative divisions in urban regions with big populations, while eup or myeon is typically the divisions in places with smaller populations. Household income level was categorized into quartiles based on average monthly income. Education was categorized into the following categories: elementary school or below, middle school, high school, or college graduation and above. Occupation was categorized into three categories: white color, blue color or unemployed. Marital status was categorized as unmarried or married.

To evaluate behavioral health characteristics, our study examined smoking, alcohol consumption, BMI, sleep duration, self-rated health, fatigue and stress. Smoking status was categorized as being a current smoker or a non-smoker at the time of the survey assessment. Alcohol consumption was categorized as ≤1 drink per week or ≥2 drinks per week. BMI was employed as a continuous variable obtained from an individual’s weight and height (kg/m^2^). Sleep duration was recorded as the average daily sleep time. Self-rated health was classified as good, moderate, or poor. Fatigue and stress were classified as low or high.

### Statistical analysis

All data used in this study were statistically processed using SPSS statistical software (version 25; SPSS Inc., Chicago, IL, USA). For continuous variables, we calculated means and standard deviations and compared differences between each group using a two-sample t-tests. For nominal variables, the frequency of each item was calculated and chi-square tests were performed to determine differences between the comparison and control groups. Logistic regression analyses were performed for variables showing a significant difference (p<0.05) between TMD groups and controls. As a result, we obtained odds ratios (ORs) and 95% confidence intervals (CIs) for prevalence of TMD according to comorbidities. In the logistic regression analysis, the adjusted variables were town, education, occupation, marital status, smoking, stress, fatigue and self-rated health status.

### Ethics statement

KNHANES-V was conducted by the KCDC, and informed consent was obtained from all subjects participating in this study. The study protocol was approved by the institutional review board (IRB) of the KCDC (approval numbers: 2010-02CON-21-C, 2011-02CON-06-C, 2012-01EXP-01-2C). This research information is available online as an open-source resource published by KNHANES. The data use permission protocol was completed by submitting a usage agreement when downloading the data. This study was conducted with permission from the IRB committee of Kyung Hee University Hospital at Gangdong Seoul, Korea (approval number: 2022-05-023).

## Results

### General characteristics

Demographic and psychosocial variables within the study population are shown in Tables 1 and 2. Participants who experienced TMD were more likely to be younger (among both men and women). Within the same age group, women with TMD were 1.3 times more likely than men with TMD. Prevalence of TMD in men was as follows: 271 (19.5%) in those aged 19–34 years, 245 (11.3%) in those aged 35–49 years, 139 (6.5%) in those aged 50–64 years, and 107 (5.8%) in those aged 65–80 years. In contrast, 509 (24.9%) of women with TMD were aged 19–34 years, 393 (13.7%) were aged 35–49 years, 245 (8.6%) were aged 50–64 years, and 198 (8.0%) were aged 65–80 years.

**Table 1:** General characteristics of study population in men.

Factors		Men 19–34			Men 35–49			Men 50–64			Men 65–80		
		TMD (-)	TMD (+)		TMD (-)	TMD (+)		TMD (-)	TMD (+)		TMD (-)	TMD (+)	
		N (%)	N (%)	*P-*value	N (%)	N (%)	*P-*value	N (%)	N (%)	*P-*value	N (%)	N (%)	*P-*value
Total		1116 (80.5)	271 (19.5)		1916 (88.7)	245 (11.3)		1984 (93.5)	139 (6.5)		1747 (94.2)	107 (5.8)	
**Age (yr)**	Mean (± S.D)	27.6 (4.5)	26.9 (4.3)	0.0123	41.7 (4.2)	40.7 (4)	0.0003	57 (4.3)	56.6 (4.3)	0.2838	72.1 (4.6)	71.9 (4.6)	0.6784
**BMI (kg/m2)**	Mean (± S.D)	24 (3.7)	24.1 (3.7)	0.7945	24.5 (3.1)	23.9 (3.2)	0.0037	24.1 (2.8)	24.5 (2.9)	0.1007	23.2 (2.9)	23.1 (3.1)	0.8228
**Sleep duration (hr)**	Mean (± S.D)	6.9 (1.2)	6.9 (1.3)	0.8660	6.8 (1.1)	6.7 (1.1)	0.5790	6.9 (1.3)	6.7 (1.4)	0.2197	6.7 (1.6)	6.5 (1.8)	0.2032
**Town**													
	Dong	994 (89.1)	237 (87.5)	0.4506	1609 (84)	209 (85.3)	0.5919	1495 (75.4)	107 (77)	0.6668	1190 (68.1)	74 (69.2)	0.8223
	Eup/myeon	122 (10.9)	34 (12.5)		307 (16)	36 (14.7)		489 (24.6)	32 (23)		557 (31.9)	33 (30.8)	
**House income**													
	Low	83 (7.5)	28 (10.4)	0.2392	121 (6.4)	10 (4.1)	0.4296	227 (11.6)	18 (13.4)	0.2533	797 (46.3)	54 (50.9)	0.2945
	Middle-low	306 (27.6)	64 (23.7)		467 (24.6)	54 (22.4)		464 (23.7)	40 (29.9)		490 (28.5)	34 (32.1)	
	Middle-high	380 (34.3)	88 (32.6)		674 (35.5)	90 (37.3)		555 (28.3)	30 (22.4)		256 (14.9)	11 (10.4)	
	High	338 (30.5)	90 (33.3)		637 (33.5)	87 (36.1)		713 (36.4)	46 (34.3)		179 (10.4)	7 (6.6)	
**Education**													
	Elementry school	0 (0)	0 (0)	0.0388	40 (2.1)	2 (0.8)	0.1423	421 (21.5)	26 (18.8)	0.6511	766 (45.2)	55 (51.4)	0.2261
	Middle school	22 (2)	6 (2.2)		94 (5)	6 (2.5)		402 (20.5)	29 (21)		296 (17.5)	12 (11.2)	
	High school	532 (48.5)	153 (56.9)		710 (37.8)	92 (37.7)		673 (34.4)	54 (39.1)		387 (22.9)	28 (26.2)	
	College	544 (49.5)	110 (40.9)		1035 (55.1)	144 (59)		461 (23.6)	29 (21)		244 (14.4)	12 (11.2)	
**Occupation**													
	White color	534 (49.2)	132 (49.3)	0.2863	1134 (60.6)	165 (67.6)	0.1030	661 (34)	45 (32.6)	0.3351	115 (6.8)	10 (9.3)	0.3478
	Blue color	248 (22.9)	51 (19)		642 (34.3)	70 (28.7)		957 (49.2)	63 (45.7)		613 (36.3)	43 (40.2)	
	Unemployed	303 (27.9)	85 (31.7)		94 (5)	9 (3.7)		328 (16.9)	30 (21.7)		963 (56.9)	54 (50.5)	
**Marital state**													
	Married	368 (33)	67 (24.7)	0.0086	1759 (91.9)	218 (89)	0.1282	1954 (98.6)	135 (97.8)	0.4713	1740 (99.7)	107 (100)	0.5436
	Unmarried	748 (67)	204 (75.3)		156 (8.1)	27 (11)		28 (1.4)	3 (2.2)		6 (0.3)	0 (0)	
**Smoking**													
	No	556 (50.7)	141 (52.2)	0.6505	912 (48.3)	118 (48.2)	0.9667	1223 (62.4)	83 (60.1)	0.5977	1281 (75.9)	78 (72.9)	0.4841
	Yes	541 (49.3)	129 (47.8)		976 (51.7)	127 (51.8)		737 (37.6)	55 (39.9)		407 (24.1)	29 (27.1)	
**Drinking**													
	≤1 per week	769 (71.7)	185 (70.6)	0.7177	1062 (58.3)	138 (58)	0.9361	997 (53.4)	65 (49.6)	0.3981	929 (61.6)	65 (67.7)	0.2293
	≥2 per week	303 (28.3)	77 (29.4)		761 (41.7)	100 (42)		869 (46.6)	66 (50.4)		580 (38.4)	31 (32.3)	
**Health status**													
	Good	539 (49.1)	104 (38.7)	0.0039	739 (39.3)	69 (28.3)	0.0037	700 (35.8)	34 (24.6)	0.0017	550 (32.4)	23 (21.5)	0.0480
	Moderate	473 (43.1)	133 (49.4)		960 (51.1)	147 (60.2)		926 (47.3)	66 (47.8)		715 (42.1)	49 (45.8)	
	Poor	86 (7.8)	32 (11.9)		180 (9.6)	28 (11.5)		332 (17)	38 (27.5)		434 (25.5)	35 (32.7)	
**Fatigue**													
	Low	1030 (93.6)	250 (92.9)	0.6766	1768 (94)	225 (92.6)	0.3933	1893 (96.4)	121 (88.3)	0.0000	1632 (94.8)	95 (88.8)	0.0079
	High	70 (6.4)	19 (7.1)		113 (6)	18 (7.4)		70 (3.6)	16 (11.7)		89 (5.2)	12 (11.2)	
**Stress**													
	Low	792 (72.1)	178 (65.9)	0.0467	1325 (70.2)	150 (61.2)	0.0043	1585 (80.9)	99 (71.7)	0.0092	1471 (87.1)	88 (82.2)	0.1457
	High	307 (27.9)	92 (34.1)		563 (29.8)	95 (38.8)		375 (19.1)	39 (28.3)		217 (12.9)	19 (17.8)	

* t-test or Chi-square test was performed to determine differences between groups with/without TMD

Abbreviations: BMI, body mass index; S.D, standard deviation

**Table 2:** General characteristics of study population in women.

Factors		Women 19–34			Women 35–49			Women 50–64			Women 65–80		
		TMD (-)	TMD (+)		TMD (-)	TMD (+)		TMD (-)	TMD (+)		TMD (-)	TMD (+)	
		N (%)	N (%)	*P-*value	N (%)	N (%)	*P-*value	N (%)	N (%)	*P-*value	N (%)	N (%)	*P-*value
Total		1536 (75.1)	509 (24.9)		2473 (86.3)	393 (13.7)		2603 (91.4)	245 (8.6)		2279 (92)	198 (8)	
**Age (yr)**	Mean (± S.D)	27.8 (4.6)	26.7 (4.7)	0.0000	41.7 (4.3)	40.6 (4.2)	0.0000	56.7 (4.4)	56.1 (4.4)	0.0293	72.5 (4.8)	72.5 (4.8)	0.9196
**BMI (kg/m2)**	Mean (± S.D)	21.8 (3.6)	21.4 (3.8)	0.0188	23.1 (3.4)	23.1 (3.6)	0.7856	24.3 (3.2)	23.5 (3.1)	0.0002	24.2 (3.4)	24.2 (3.4)	0.9822
**Sleep duration (hr)**	Mean (± S.D)	7.3 (1.4)	7.3 (1.3)	0.6734	6.9 (1.1)	6.9 (1.3)	0.8214	6.6 (1.3)	6.7 (1.5)	0.7262	6.3 (1.8)	6.3 (1.8)	0.5860
**Town**													
	Dong	1357 (88.3)	459 (90.2)	0.2564	2115 (85.5)	346 (88)	0.1833	2026 (77.8)	190 (77.6)	0.9190	1522 (66.8)	117 (59.1)	0.0282
	Eup/myeon	179 (11.7)	50 (9.8)		358 (14.5)	47 (12)		577 (22.2)	55 (22.4)		757 (33.2)	81 (40.9)	
**House income**													
	Low	101 (6.7)	30 (5.9)	0.2188	160 (6.5)	25 (6.4)	0.2910	441 (17.1)	33 (13.8)	0.3870	1228 (54.9)	113 (57.7)	0.8586
	Middle-low	413 (27.2)	116 (23)		642 (26.2)	85 (21.9)		715 (27.8)	67 (28)		510 (22.8)	44 (22.4)	
	Middle-high	518 (34.1)	181 (35.8)		800 (32.7)	140 (36)		669 (26)	59 (24.7)		277 (12.4)	22 (11.2)	
	High	486 (32)	178 (35.2)		848 (34.6)	139 (35.7)		748 (29.1)	80 (33.5)		223 (10)	17 (8.7)	
**Education**													
	Elementry school	5 (0.3)	0 (0)	0.3856	81 (3.3)	7 (1.8)	0.0422	1042 (40.6)	100 (41)	0.4277	1778 (81.9)	158 (81)	0.4997
	Middle school	22 (1.5)	4 (0.8)		158 (6.5)	18 (4.6)		568 (22.1)	46 (18.9)		189 (8.7)	14 (7.2)	
	High school	631 (41.7)	214 (42)		1184 (48.5)	177 (45.5)		709 (27.6)	68 (27.9)		163 (7.5)	20 (10.3)	
	College	856 (56.5)	291 (57.2)		1017 (41.7)	187 (48.1)		246 (9.6)	30 (12.3)		41 (1.9)	3 (1.5)	
**Occupation**													
	White color	713 (47.1)	258 (50.7)	0.3773	984 (40.3)	168 (43.2)	0.0063	652 (25.4)	51 (20.9)	0.1575	96 (4.4)	6 (3.1)	0.6714
	Blue color	72 (4.8)	22 (4.3)		360 (14.8)	34 (8.7)		703 (27.4)	63 (25.8)		509 (23.5)	47 (24.1)	
	Unemployed	728 (48.1)	229 (45)		1095 (44.9)	187 (48.1)		1213 (47.2)	130 (53.3)		1565 (72.1)	142 (72.8)	
**Marital state**													
	Married	755 (49.2)	188 (36.9)	0.0000	2384 (96.4)	370 (94.1)	0.0287	2572 (98.9)	243 (99.2)	0.6668	2266 (99.6)	198 (100)	0.4032
	Unmarried	781 (50.8)	321 (63.1)		88 (3.6)	23 (5.9)		29 (1.1)	2 (0.8)		8 (0.4)	0 (0)	
**Smoking**													
	No	1406 (92.5)	449 (88.2)	0.0028	2321 (95.2)	363 (93.1)	0.0764	2473 (96.3)	229 (93.9)	0.0546	2094 (96.6)	190 (96.9)	0.7941
	Yes	114 (7.5)	60 (11.8)		117 (4.8)	27 (6.9)		94 (3.7)	15 (6.1)		74 (3.4)	6 (3.1)	
**Drinking**													
	≤1 per week	1275 (87.9)	431 (88.1)	0.8747	1940 (87.7)	325 (88.8)	0.5667	1745 (91.8)	173 (91.1)	0.7236	1101 (92.1)	113 (94.2)	0.4253
	≥2 per week	176 (12.1)	58 (11.9)		271 (12.3)	41 (11.2)		156 (8.2)	17 (8.9)		94 (7.9)	7 (5.8)	
**Health status**													
	Good	598 (39.5)	147 (28.9)	0.0000	904 (37)	107 (27.5)	0.0000	715 (27.8)	40 (16.3)	0.0000	479 (21.9)	21 (10.8)	0.0003
	Moderate	736 (48.6)	267 (52.5)		1247 (51.1)	206 (53)		1263 (49.1)	123 (50.2)		866 (39.6)	77 (39.5)	
	Poor	180 (11.9)	95 (18.7)		289 (11.8)	76 (19.5)		595 (23.1)	82 (33.5)		842 (38.5)	97 (49.7)	
**Fatigue**													
	Low	1280 (93.2)	417 (89.3)	0.0061	2221 (93.5)	336 (89.1)	0.0023	2386 (93.1)	223 (91.8)	0.4535	2098 (93.8)	180 (91.4)	0.1844
	High	93 (6.8)	50 (10.7)		155 (6.5)	41 (10.9)		178 (6.9)	20 (8.2)		139 (6.2)	17 (8.6)	
**Stress**													
	Low	970 (63.8)	291 (57.2)	0.0075	1803 (74)	251 (64.4)	0.0001	1940 (75.6)	180 (73.8)	0.5317	1577 (72.8)	126 (64.9)	0.0188
	High	550 (36.2)	218 (42.8)		635 (26)	139 (35.6)		627 (24.4)	64 (26.2)		588 (27.2)	68 (35.1)	

* t-test or Chi-square test was performed to determine differences between groups with/without TMD

Abbreviations: BMI, body mass index; S.D, standard deviation

Among patients with TMD, the mean BMI was significantly lower in men aged 35–49 years, women aged 19–34 and 50–64 years than those without TMD. In all groups, the prevalence of TMD was significantly higher in the group responding as moderate or poor than in the case of good health status. Also, the prevalence of TMD was significantly higher in men aged 50–64 years, 65–80 years, women aged 19–34 and 65–49 years who responded that fatigue was high. Stress also showed a high rate of response when TMD was present in all groups, and there was a statistically significant difference in 6 groups except for men aged 65–80 years and women aged 50–64 years.

The following variables differed according to the presence of TMD only in certain groups: town (women aged 65–80 years), education (men aged 19–34 years, women aged 35–49 years), occupation (women aged 35–49 years), marital state (men aged 19–34 years, women aged 19–49 years), smoking (women aged 19–34 years).

### Medical comorbidities

**Fig 2:**
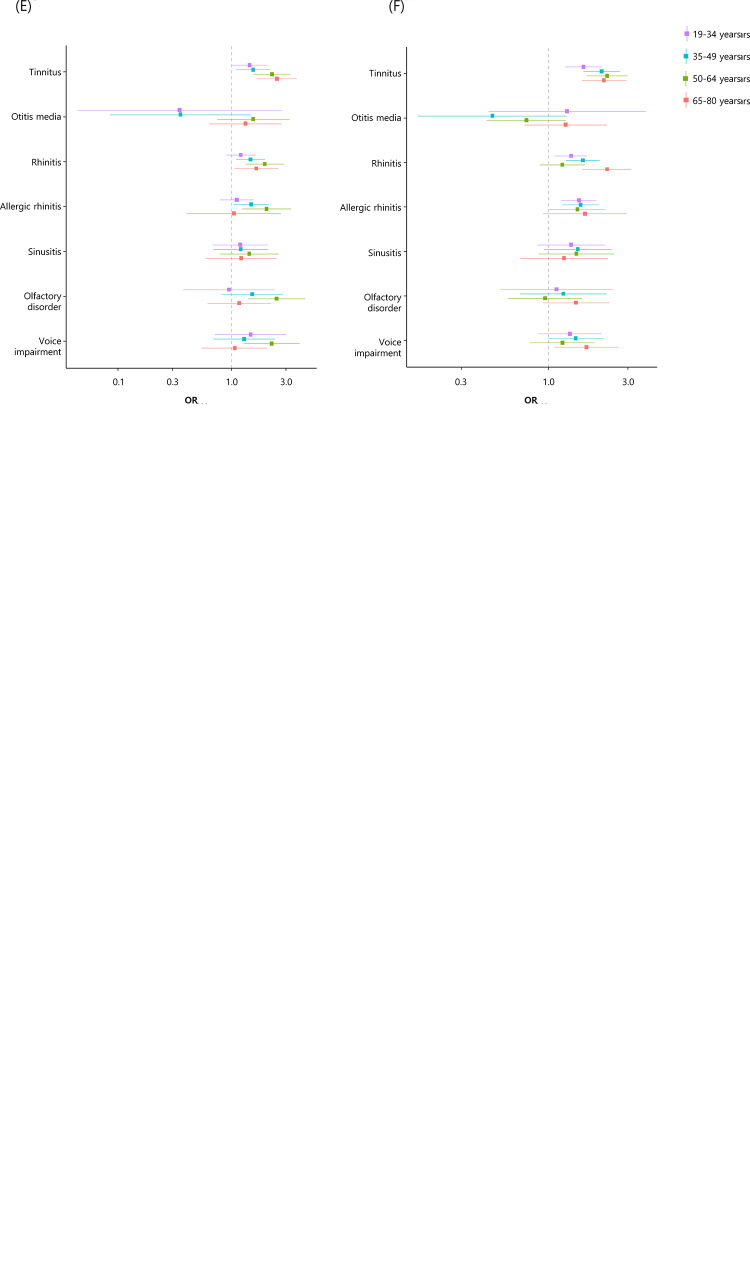
ORs for prevalence of TMD associated with medical comorbidities. This model was adjusted for town, education, occupation, marital status, smoking, stress, fatigue and health status in estimation of ORs and 95% C.I. The y-axis variables in (A)-(D) are chronic diseases, and those in (E), (F) are otolaryngological symptoms. The ORs for (A), (C) and (E) were obtained in men groups, and those for (B), (D) and (F) were obtained in women groups.

Fig 2. displays the ORs for prevalence of TMD associated with chronic diseases and otolaryngological symptoms in men and women.

The results of chi-square tests between the presence or absence of medical comorbidities and TMD can be found in S1 File. In addition, two type of logistic regression model that calculated the ORs for prevalence of TMD associated with medical comorbidities are attached as a S2 Table in S1 File. In the case of adjusted model among them, the ORs for TMD are visualized and shown in Fig 2.

#### Associations between chronic diseases and TMD

As shown in Fig 2(A)–2(D) and S 2, there was no chronic disease in which the ORs for prevalence of TMD showed significant values for the entire group. However, depending on the type of chronic disease, the ORs of prevalence of TMD was significant in several groups, or the value was significant only in one or two specific groups. When accompanied by CHHF (complaining of cold hands and feet even in the normal temperature range), the ORs for prevalence of TMD was greater than 1 in men aged 19–34, 35–49, 65–80 years and women aged 35–49 years. The OR for prevalence of TMD was 1.60 (95% C.I, 1.14–2.25) in men aged 35–49 years, 1.44(95% C.I, 1.14–1.81) in women aged 35–49 years and 1.43 (95% C.I, 1.07–1.90) in women aged 50–64 years with migraine. Participants who experienced depression were more likely to have TMD (OR 1.68, 95% C.I 1.00–2.83 in men aged 50–64 years; OR 1.39, 95% C.I 1.05–1.85 in women aged 35–49 years; OR 2.01, 95% C.I 1.46–2.77 in women aged 65–80 years). For the following chronic diseases, the ORs for prevalence of TMD was significantly observed in only one group: hypertension in men aged 50–64 years (OR 0.62, 95% C.I 0.41–0.94), ischemic heart disease in men aged 35–49 years (OR 4.38, C.I 1.54–12.47), diabetes mellitus in men aged 35–49 years (OR 0.21, C.I 0.05–0.88), osteoarthritis in women aged 50–64 years (OR 1.38, 95% C.I 1.03–1.86).

#### Associations between otolaryngological symptoms and TMD

Fig 2(E) and 2(F) display the ORs for prevalence of TMD associated with otolaryngological symptoms for each group. Tinnitus was the only otolaryngology symptom in which the ORs for prevalence of TMD were greater than 1 in all groups. When participants responded to the presence of rhinitis, the ORs for prevalence of TMD were greater than 1 for the 6 groups (men aged 35–49,50–64, 65–80 years, women aged 19–34,35–49 and 65–80 years). In the case of allergic rhinitis, although slightly different from the results of rhinitis, the ORs for prevalence of TMD were found to be greater than 1 in 5 groups (men aged 35–49, 50–64 years, women aged 19–34, 35–49 and 50–64 years). With regard to other otolaryngological symptoms, the ORs for prevalence of TMD were as follows: olfaction disorder (OR 2.49, 95% CI 1.39–4.43 in men aged 50–64 years) and voice disorder (OR 2.25, 95% CI 1.28–3.96 in men aged 50–64 years, OR 1.69, 95% CI 1.09–2.63 in women aged 65–80 years).

## Discussion

### Chronic diseases and TMD

#### Hypertension and diabetes mellitus

Hypertension are rarely reported in relation to TMD, in contrast to headache, tinnitus, and depression, which are regarded as major comorbidities of TMD. In Fig 2(A), the ORs for prevalence of TMD was lower than 1 only in men with hypertension aged 50–64 years, but it was difficult to find sufficient evidence to explain this result. However, the report that patients with orthostatic hypotension tend to have TMD due to problems with autonomic nerve control can indirectly support the above results [15].

On the other hand, the OR for prevalence of TMD in patients with diabetes mellitus (DM) was 0.21, showing a significant difference only in men aged 35–49 years. This result contradicts the previous report that DM causes an inflammatory response and affects TMJ to be involved in pathogenesis. There is still insufficient evidence to discuss the association between hypertension, diabetes, and TMD, and more research is needed in the future.

#### Ischemic heart disease

Risk factors for Ischemic heart disease include high blood pressure, smoking, hyperlipidemia, diabetes, obesity, and family history [16]whereas the relationship between TMD and ischemic heart disease is not well known. Although few studies mention the association between them, in Marcelo’s review, 38% of 186 patients with ischemic heart disease experienced not only chest pain, but also TMJ pain, arm pain and shoulder pain. Moreover, 6% complained of only orofacial pain without any symptoms of chest pain [17]. In one report, a 48-year-old man visited the dentist with sudden jaw and tooth pain, but no clear cause was found. Suspecting the pain were related to heart disease, the dentist requested a consultation with a cardiologist and the patient was diagnosed with angina pectoris as a result. Following cardiovascular surgery, the patient reported that the pain in his teeth and jaw had disappeared [18]. As the background of this phenomenon, the authors mention the spinothalamic tract cells of C2 and C3, where cardiac nociceptive signals are transmitted via vagus nerve and converged with nerves responsible for pain in the jaw and teeth [19]. In our study, ORs for prevalence of TMD in reference to myocardial infarction were only significant in certain groups of men (OR 7.58 in men aged 35–49 years; OR 2.79 in men aged 50–64 years). This fact can be interpreted as evidence that strongly casts into doubt whether TMD symptoms with no specific cause in middle-aged men are related to heart disease.

#### Osteoarthritis

A study conducted in 8,964 people diagnosed with TMD (among a total of 189,977 adults) found that 83% of the TMD patients experienced pain elsewhere in the body and that 62.4% reported pain or stiffness mainly in other joints. These rates were significantly different from those detected in the group without TMD and were found to be more pronounced in women [20]. In addition, Bonato et al. reported that, among 337 Brazilians (68.5% women), those with TMD were 5.5 times more likely to report joint pain in other areas than the control group [8]. Trost et al. reported that knee pain (OR 1.40, 95% CI 1.14–1.72), hand pain (OR 1.81, 95% CI 1.39–2.37), and hip pain (OR 2.13, 95% CI 1.62–2.79) were detected in women with TMJ pain. However, the OR for men was not significant. In addition, in both men and women with TMJ pain, the number of regions in the body where patients complained of pain was significantly higher than that in the group without TMJ pain, and it was speculated that TMJ pain could be regarded as a factor within the generalized pain phenotype [21].

Through the studies introduced above, patients with TMD (especially women) have a high rate of accompanying pain in other joints, which can be inferred to be related to the development of TMD. The fact that a 1.38-fold increase in prevalence of TMD was observed in the 50-64year group of women diagnosed with arthritis in Fig 2(D) indirectly supports the above inference. Of course, it is not enough to confirm the association between arthritis and TMD because the mentioned study examples were based on joint pain rather than arthritis in other parts of the body.

A previous study investigated TMD symptomology according to the presence or absence of knee osteoarthritis (knee OA). Compared with the control group (n = 150), patients with knee OA(n = 144) exhibited a small range of motion and large amount of joint noise. However, there was no difference in discomfort during palpation or pain during opening between the two groups [22].

A recent study by Wang et al. reported that TMJ and knee OA share a common feature of chronic degenerative changes in soft and hard tissues around the joint. However, TMJ OA mainly occurs in young women, and knee OA occurs mainly in postmenopausal women. This is because estrogen has different effects on each disease through estrogen-related receptors [23]. Structurally, the joint disc of TMJ is a fibrocartilage composed of collagen I, and the knee joint is composed of hyaline cartilage composed of collagen II. In knee OA, estrogen-related receptor-α (ERR-α) inhibition, inflammation, and ERR-γ overexpression increase metalloproteinases-3,9, hence leading to OA [24, 25]. On the other hand, in TMJ arthritis, estradiol stimulation leads to the loss of the extracellular matrix through the estrogen-ERRβ-hypoxia-inducible factor 2-α pathway, hence leading to arthritis [26]. Although estrogen is an important factor within various types of arthritis, it cannot be confirmed with certainty that a patient with temporomandibular arthritis has a high probability of developing other types of arthritis as estrogen is involved in different mechanisms depending on the joint area.

#### Migraine

According to Fig 2(B) and 2(D), an OR >1 for TMD occurring with migraine was observed in men aged 35–49 years and women aged 35–64 years. Many studies related to TMD and migraine have been published in recent years. In a previous study, the OR for migraine in patients with painful TMD was 1.58, and in these cases, the frequency of migraine was higher than in the control group (OR 1.93; 95% CI 1.27 to 2.93; p<0.01), and more drugs were prescribed (OR 2.37; 95% CI 1.43 to 3.92; p<0.01) [27]. In another study, the prevalence of headache in non-TMD patients was 15.2%. However, in TMD patients, the prevalence of headache was 27.4%, and headache was more closely associated with myogenous TMD than with arthrogenous TMD. Conversely, 56.1% of patients with headache presented with TMD, and this rate increased when the headache was a migraine or tension headache and was likewise higher in women overall [28].

Cutaneous allodynia, one of the common symptoms of migraine, occurs in only approximately 40% of migraine patients without TMD, in approximately 86.9% of patients with myofascial TMD, and in approximately 84.0% of patients with myofascial and arthrogenous TMD. In evaluating disability in migraine patients, researchers detected a correlation with higher scores as well as higher severity when TMD was present as compared to these metrics in non-TMD migraine patients [29]. Moreover, when TMD and migraine cooccurred, the combination of both treatments significantly reduced the number of days of migraine as well as the severity of migraine as compared to a single disease-specific treatment for one disease only. Referring to the OPPERA prospective cohort study, a longitudinal and large-scale of TMD investigation, the annual incidence rate of TMD was 3.84 in patients with migraine, and was computed 5.31 in patients with tension-type headache compared to 2.91 in control group [30].

Both the prevalence and incidence rate of TMD rose in that research as the frequency and severity of headache increased, confirming previous small sample studies’ findings.

Taken together, the above studies suggest that migraine and TMD show bidirectional effects, with the presence of one of these diseases increasing the prevalence of the other disease, and that these diseases may share a common pathophysiological mechanism [31]. First, both diseases involve the same nociceptive system via a “trigeminocervical complex.” More specifically, nociceptive information from the periphery of the head and mandible converges to the caudal nucleus of the trigeminus and involves specific central pathways related to pain control in the thalamus, brainstem nuclei, sensitive cortex, and limbic system. Both diseases can cause craniofacial allodynia, and both present with associated pain symptomology. In addition, both diseases can induce craniofacial allodynia and referred pain, with peripheral and central sensitization involved in the background [28]. Another plausible theory suggests that these diseases may share a mechanism of abnormal autonomic cardiovascular regulation [32]. More specifically, migraine is associated with autonomic dysfunction. In a previous study, it was assumed that the activation of the vagus nerve that occurs after clenching for 5 s differs between migraine patients and healthy people. To measure this, heart rate variability and blood pressure changes were observed. As a result, a significant difference in high-frequency intensity changes reflecting vagus nerve activity was detected in migraine patients, indicating that the trigemino-cardiac reflex was enhanced compared to that in normal subjects. Recently, CGRP, a neuropeptide that is thought to be essential for inducing migraine, has been implicated in the pathogenesis of TMD and cranial hypersensitivity and has been suggested as a target within TMD treatment [33].

#### CHHF

CHHF are symptoms of a type of systemic circulation disorder caused by vasomotor changes and are related to autonomic dysfunction. This disease, which is easily confused with Raynaud’s syndrome (a disease that manifests as cold/blue hands and feet), is associated with various conditions, including hypotension, anemia, hormonal abnormalities, and menopausal symptoms.

Kong et al. reported that women aged 19–59 years presenting with CHHF (n = 70) had a weaker and thinner body type and were more vulnerable to fatigue, indigestion, dry mouth, and skin than controls (n = 68) [34]. In another study limited to women in their 20s, in addition to sensitivity to cold stimulation, the following characteristics were noted in patients presenting with CHHF: sleep disturbance, irregularities in circadian body rhythm, white finger phenomenon, numbness of limbs, and stiffness in hands [35].

The present study confirmed the association between CHHF and TMD in women aged 35–49 years as well as in most age groups in men (except in men aged 50–64 years). Patients with TMD had a lower mean BMI, higher stress and fatigue levels, and lower self-rated health evaluations than normal subjects, which can be seen as common features with the aforementioned patients with CHHF (although these findings were limited to female patients).

To date, no prior studies have reported on the relationship between TMD and CHHF. However, there are some observations that can be indirectly inferred. For example, hypersensitivity to stimulation with cold and warmth has been observed in patients with IBS, which is closely related to chronic TMD due to peripheral and central sensitization.

#### Depression

In this study, responses with regard to negative emotional variables (stress, fatigue, depression) were closely related to the overall prevalence of TMD. Notably, in women aged 35–49 years and in men aged 50–64 years, all emotional variables were correlated with TMD, and these two groups had the highest number of comorbidities related to prevalence of TMD. Thus, it can be inferred that there exists a close relationship between TMD, chronic diseases, and emotional variables. Considering the negative effects of various emotional influences (including stress) on all systemic diseases, the above results seem reasonable. On the other hand, in women aged 50–64 years (unlike in other women groups), no significant associations were found with any emotional variables in our study. Although there have been reports that TMD is highly associated with emotional problems, especially in women, the results of this study are noteworthy because no previous studies have compared the influence on emotional variables according to both age and sex.

The hypothesis that TMD and psychosocial factors are pathologically related has been proposed since the early 1980s. There is an interdependence between TMD and psychosocial variables, and the importance of the emotional perspective in TMD treatment cannot be overemphasized when examining this multidirectional relationship [36]. The AXIS-II protocol for Diagnostic Criteria for Temporomandibular Disorders, commonly used in TMD diagnosis, is a diagnostic tool that reflects this perspective. Moreover, it is known that emotional and physiological changes caused by stress accelerate the development of somatic disease.

To date, many papers have been published addressing the relationship between TMD and somatization. Somatization is highly correlated with depression and anxiety, which are in turn closely related to stress responses [37, 38]. TMD patients have been found to present with an up-regulated hypothalamic-pituitary-adrenal *(*HPA) axis, higher cortisol secretion, and higher depression, anxiety, and pain catastrophizing scores than controls [39]. In addition to the activation of the HPA axis, the sympatho-adrenal medullary (SAM) system is also triggered during stress; moreover, an “exaggerated and blunted” SAM system and HPA axis reactivity have been found to predict future mental/physical health and illness outcomes, including depression, anxiety, and musculoskeletal pain [40]. Moreover, the effects of stress on pain have been found to be modified by genetic variation, more specifically by variations in the gene encoding catechol O-methyltransferase [9].

We note that sustained emotional tension leads to increased masticatory muscle activity and abnormal functional activity. Stress, anxiety, and specific personality traits predict an increased frequency of parafunctional teeth contact. In other words, an individual’s emotional reactivity/instability can increase onset probability with regard to TMD. Conversely, even when TMD has already progressed, an unstable emotional state leads to the worsening and continuation of symptoms. In patients with chronic TMD pain, the intensity, duration, and extent of pain depend on the concurrent state of anxiety, depression, and somatic symptomology [41]. Patients with depression have more pain intensity or functional limitations than those without depression and show lower efficacy with regard to pain treatments.

In a previously published review addressing the common aspects of chronic fatigue syndrome and TMD, 20–50% of cases were found to cooccur. It was presumed that this was due to a common central mechanism. In the seven papers covered in the review, the sex distribution was 60–90% female, the average age was 32–41 years, and relatively young patients were included. The TMD OR was consistent with the high [37].

### Otolaryngological symptoms and TMD

#### Tinnitus

In the present study, significant ORs for prevalence of TMD were observed in all groups. Tinnitus, which presents with symptoms of ringing in the ears, is a heterogeneous condition that is not attributed to only one cause. Therefore, the clinical management of tinnitus may vary depending on the specific subtype79. Compared to the prevalence of tinnitus in patients without TMD, which ranges from approximately 15% to 30%, the prevalence of tinnitus is more than twice in patients with TMD 80,81

In common tinnitus, the comorbidity rate with TMD is roughly 19%. However, in severe tinnitus, this rate has been found to increase to 36%, suggesting that TMD contributes to the severity of tinnitus. Moreover, in a group with tinnitus accompanying TMD, tinnitus was controlled by mandibular and head movements, hence supporting the somatic modulation hypothesis82. In addition, fusiform cells in the dorsal cochlear nucleus integrate auditory and somatosensory input from the head and neck. The activity of these cells was confirmed in a noise-induced tinnitus animal model. On the other hand, in a noise-induced tinnitus condition, an experiment in which sound and transcutaneous electric stimulation were simultaneously applied to the skin layer of the trigeminal nerve and to cervical nerve endings showed that tinnitus was significantly alleviated by plastic changes in fusiform cells as compared to each of the above stimulations administered alone83. Therefore, considering that TMD is closely related to the sensitization and dysfunction of the somatosensory input system of the trigeminal nerve, TMD treatment can be expected to alleviate the symptoms of tinnitus.

Moreover, the remarkable similarity between tinnitus and chronic pain-related pathways also supports the theory that TMD is an important variable in tinnitus, considering that TMD is a chronic pain disease84. In addition, it has been reported that sex does not affect the incidence of somatic tinnitus. Taken together, this evidence suggests that TMD and tinnitus have been found to exert mutual effects, and neither disease appears to be significantly associated with age and sex.

#### Rhinitis, allergic rhinitis

In the KNHANES survey, which is the basis of this paper, the items of otolaryngology included not only rhinitis but also the presence or absence of allergic rhinitis. Therefore, our study was able to obtain OR for each with respect to prevalence of TMD. As a result, ORs for prevalence of TMD were greater than 1 in patients with symptoms of rhinitis or allergic rhinitis, regardless of gender and age.

In the previous literature, studies related to allergic rhinitis and TMD were mainly presented. Allergic rhinitis is one of the atopic diseases, characterized by an exaggerated immune response to an antigen.

Recently, a study was published on the relationship between atopic disease and five chronic pain conditions (TMD, headache, irritable bowel syndrome, LBP, and fibromyalgia) [42]. The authors predicted that sleep problems and fatigue, considered common symptoms among these five overlapping pain conditions, would also affect atopic diseases. More specifically, itchy skin or difficulty breathing (i.e., symptoms occurring within atopic disease) interfere with sleep, and poor sleep quality lowers a patient’s physical condition, thereby creating negative feedback that slows the improvement of the disease. Moreover, if chronic pain, sleep, and fatigue are already worsened, this can promote the onset of atopic disease. Among the 660 subjects included in the profiled study, the average number of atopic diseases that were comorbid with TMD (in 180 patients) was 1.89, whereas the average number of accompanying atopic diseases in the control group (463 patients) was 1.06, indicating a significant difference between the groups.

#### Olfaction disorder

A notable finding related to olfaction disorder in our study was that only men aged 50–64 years showed an elevated (2.69-fold) increased risk of prevalence of TMD. However, although few studies have directly mentioned the relationship between olfaction disorder and TMD, some studies have indirectly inferred the relationship between these diseases.

In a study examining the sensory characteristics (gustative, olfaction, thermal, mechanical, and pain stimuli) in patients with chronic facial pain (trigeminal neuralgia, TMD, BMS, and idiopathic facial pain) over the course of more than 6 months, trigeminal neuralgia patients had a higher olfactory threshold than controls. However, this study was limited in that mention of TMD and olfactory-related data were omitted and the patient sample was small, ranging from 8 to 29 patients in each group [43].

In a prior study examining the relationship between taste and TMD, the control group (n = 2,026) experienced a taste disorder rate of 2%, whereas the corresponding rate was 6% in patients with TMD (n = 301) and TMD pain intensity was found to increase by 29% for every 1% increase in the frequency of taste disturbance. Moreover, it was speculated that, similar to other chronic pain conditions, central mediation is involved in the pain mechanisms underlying TMD. In fact, in a previous study, an enhanced response to other sensory stimuli that was not mediated by the trigeminal nerve was observed in patients with TMD. In addition, in a study reporting similar results, patients with chronic LBP and complex musculoskeletal pain had higher scores with regard to general taste concentration than the control group [44].

We note that Hummel reviewed the interaction between the trigeminal nerve and olfactory system in the nasal cavity from a neuroanatomical perspective. In patients with anosmia, there is evidence that trigeminal input is not amplified in the orbitofrontal cortex and rostral insula in the central nervous system, resulting in decreased trigeminal sensitivity. However, the increase in trigeminal reactivity at the peripheral level in patients with anorexia or olfaction disorder seems to be an adaptive mechanism that compensates for this missing central nervous system amplification signal [45].

#### Voice disorder

In this study, ORs for TMD presenting with self-cognitive voice disorder were 1.56 and 2.42 in women aged 65–80 years and in men aged 50–65 years, respectively. To date, few papers have evaluated TMD in relation to voice disability. However, a study investigating TMD in 52 Iranian women with voice-related disability found that TMD severity was positively correlated with the voice handicap index [46]. The authors concluded that tension in the head and neck could be transmitted to the larynx through the muscular connection between the mandible and hyoid bone. It was hypothesized that this tension would reduce voice range, increase fatigue, and lead to changes in voice resonance that would eventually lead to the patients’ dissatisfaction with their voice. In addition, it was observed that the change in voice quality interfered with the proper transmission of meaning and emotion, leading to secondary stress and psychosocial problems.

### Strength and limitation

As a major strength of this study, it was possible to increase the reliability of the results of the dependent variable because the groups were matched by age and sex to make them homogeneous. Previous studies that did not classify groups based on age and sex investigated the same data as this study, but in particular, there are some discrepancies from our findings in that the type of comorbidities with a high odds ratio of TMD are different. In this study, Ischemic heart disease, olfaction disorder, voice disorder, and CHHF were also proven to be linked with TMD through chi square analysis and logistic regression analyses. Since the subject group was segmented, it was enabled to confirm which comorbidities were linked with associated TMD in a given age sex group. In other words, it is a new finding that stress, depression, and tinnitus variables have a high association with TMD in the entire group, but only in middle-aged men in the case of heart disease. Given the lack of volunteers in many clinical trials, the findings of this study may be useful in identifying participants for additional TMD and comorbidities investigations. Another advantage is that the bias was minimized by selecting a sample using a stratified sampling method for a large number of subjects. In addition, because the questionnaire and examination were completed by skilled specialists, the study’s dependability was enhanced.

As a limitation of this study, since it was a result from a cross-sectional design based on a specific point in time, only the correlation, not the causal relationship, was examined. Another drawback of the study is that WHO-recommended TMD diagnostic criteria were applied in our study rather than the commonly used DC/TMD or RDC/TMD techniques, it was unable to discriminate between myogenous and arthrogenous TMD. Previous research has shown that myogenous TMD is more closely linked to comorbidities such low back pain and headache than arthrogenous TMD, however this was not verified in our investigation. Finally, the prevalence of comorbidities was determined based on the patient’s medical history rather than the doctor’s diagnosis, which is a study limitation.

## Conclusion

TMDs are diseases that need to be understood from a multilayered perspective as they are caused by the action of various pathophysiological backgrounds. This study confirmed that the types and effects of comorbidities related to the prevalence of TMD may differ depending on the patient’s age and sex and this will increase the predictability of the onset of TMD. Ultimately, if confirmed, our findings will guide screening protocols and general clinical decision making.

## Supporting information

S1 File S1 is a chi-square test table, and S2 includes two types of logistic regression analysis tables. (XLSX)
